# Evaluation of model refinement in CASP13

**DOI:** 10.1002/prot.25794

**Published:** 2019-08-20

**Authors:** Randy J. Read, Massimo D. Sammito, Andriy Kryshtafovych, Tristan I. Croll

**Affiliations:** ^1^ Department of Haematology Cambridge Institute for Medical Research, University of Cambridge Cambridge UK; ^2^ Genome Center University of California Davis California

**Keywords:** CASP, model refinement, molecular replacement, structure prediction

## Abstract

Performance in the model refinement category of the 13th round of Critical Assessment of Structure Prediction (CASP13) is assessed, showing that some groups consistently improve most starting models whereas the majority of participants continue to degrade the starting model on average. Using the ranking formula developed for CASP12, it is shown that only 7 of 32 groups perform better than a “naïve predictor” who just submits the starting model. Common features in their approaches include a dependence on physics‐based force fields to judge alternative conformations and the use of molecular dynamics to relax models to local minima, usually with some restraints to prevent excessively large movements. In addition to the traditional CASP metrics that focus largely on the quality of the overall fold, alternative metrics are evaluated, including comparisons of the main‐chain and side‐chain torsion angles, and the utility of the models for solving crystal structures by the molecular replacement method. It is proposed that the introduction of these metrics, as well as consideration of the accuracy of coordinate error estimates, would improve the discrimination between good and very good models.

## 1 INTRODUCTION

The refinement category was introduced in CASP8 to assess potential strategies for further improving the quality of some of the best models produced by existing structure prediction pipelines. Although these strategies could in principle be introduced into the pipelines that they follow on from, having a separate refinement category allows focus on the endgame when models are already reasonably accurate. It also allows the exploration of what becomes possible when significantly greater computing resources can be devoted to a smaller number of starting models.

Over the years, there have been signs of progress but there have also been recurring themes in the assessments of this category.^1^, ^2^, ^3^, ^4^, ^5^ It has always been true that, considering all submissions in total, more of the refined models become worse than the starting model rather than better. This reflects considerations that there are many degrees of freedom in the space of incorrect models, so that there are more ways to degrade a model than to improve it; the search space has many local minima with a relatively narrow convergence radius around the true structure; and many groups use this category (as well as other categories in CASP) as a way to experiment with novel ideas. As early as CASP8,^1^ it was recognized that it is much easier to improve the agreement of a model with physics (geometric criteria including torsion angles and clashes, as measured for instance by MolProbity^6^) than the overall fidelity of the fold, and that for distant models the two measures do not tend to be correlated. Because of problems with the dimensionality of the search, relatively conservative strategies that restrain shifts from at least the better parts of the starting model tend to be more successful because they avoid serious degradation of the model; as a result, the refined structures are almost always closer to the starting model than to the experimental structure.

Nonetheless, there has been real progress in this category. In CASP8,^1^ only one group (Lee) succeeded in improving the average global distance test total score (GDT_TS)^7^ from the starting models, whereas by the time of CASP12 8 of 39 groups succeeded in improving the more stringent high‐accuracy GDT_HA score.^5^

## 2 MATERIALS AND METHODS

### 2.1 Target classification

A total of 31 refinement targets were chosen, with two exceptions, from among the best server models for evaluation units from the various structure prediction categories, comprising the easy and hard versions of template based modeling (TBM), free modeling (FM) and the intermediate TBM/FM. The exceptions were the refinement models for the two subunits of target T0986, that is, R0986s1 and R0986s2; both of these models were submitted by group A7D and were substantially better than the best server models. Two targets were subsequently canceled because of unexpectedly early publication of the experimental structures, leaving 29 for evaluation (Table 1). Feedback from CASP12 suggested that refinement targets larger than about 200 residues were too demanding of computational resources, so targets were restricted to domains ranging from 59 to 204 residues. Visual inspection was used to confirm that the starting models were of reasonable quality in at least some regions of the structure, but also that there was room for improvement by refinement of aspects such as sequence register, choices of conformer, or relative orientations of subdomains or secondary structure elements.

**Table 1:** Source of refinement targets and information given to predictors

Target	Residues included	Nres	Initial category	Start model	Start GDT_HA	Additional information for predictors
R0949	43‐95, 106‐181	129	TBM/FM	TS221_1	49	Residues 1‐42, 96‐105, and 182‐183 are not ordered in the crystal structure and deleted from the starting model. The structure contains a bound Cu ion
R0957s2	7‐164	158	FM	TS498_1	39	Residues 1‐6 are absent from the target structure and deleted from the starting model
R0959	1‐189	189	TBM‐hard	TS368_1	45	
R0962	2‐178	177	TBM‐easy	TS246_1	63	Residues 1 and 179‐220 are absent from the target and deleted from the starting model
R0968s1	6‐123	118	FM	TS368_1	45	Residues 1‐5 and 124‐126 are absent from the target structure and deleted from the starting model
R0968s2	1‐116	116	FM	TS368_3	50	
R0974s1	2‐70	69	TBM‐easy	TS488_1	66	Residues 1 and 71‐72 are absent from the target structure and deleted from the starting model
R0976‐D1	9‐128	120	TBM‐easy	TS337_1	69	This refinement target corresponds to domain 1 (residues 9–128) of T0976
R0976‐D2	129‐252	124	TBM‐easy	TS337_1	65	This refinement target corresponds to domain 2 (residues 129–252) of T0976
R0977‐D2	360‐563	204	TBM‐easy	TS402_3	68	This refinement target corresponds to domain 2 (residues 360–563) of T0977. Remember that the original target is a homotrimer. The interface in the starting model is modeled reasonably accurate
R0979	6‐97	92	TBM‐hard	TS470_1o	55	This is the first oligomeric refinement target in CASP. Being a trimer, it is somewhat longer than other refinement targets in CASP13: 276 residues total. GDT_HA of the starting model's monomeric unit is 55 (on 92 residues; residues 1–5 and 98 are absent from the experimental structure). LDDT score of the oligomeric starting model is 0.81; the interchain contact accuracy score *F*1 = 43%. All rules pertaining to submission of regular homooligomeric targets apply here
R0981‐D3	191‐393	203	TBM/FM	TS261_1	32	This refinement target corresponds to domain 3 (residues 191–393) of T0981. Remember that the original target is a homotrimer. The interface in the starting model is modeled reasonably accurate
R0981‐D4	403‐513	111	TBM‐hard	TS368_1	45	This refinement target corresponds to domain 4 (residues 403‐513) of T0981. Remember that the original target is a homotrimer. The interface in the starting model is modeled reasonably accurate
R0981‐D5	514‐640	127	TBM‐hard	TS116_1	42	This refinement target corresponds to domain 5 (residues 514‐640) of T0981. Remember that the original target is a homotrimer. Residues 605‐623 are a part of a homotrimer interface and modeling of this segment can be improved the most
R0982‐D2	146‐277	132	TBM‐hard	TS324_1	50	This refinement target corresponds to domain 2 (residues 146‐277) of T0982
R0986s1	5‐92	88	TBM/FM	TS043_4	59	Residues 1–4 are absent from the target structure and deleted from the starting model
R0986s2	1‐155	155	FM	TS043_4	49	
R0989‐D1	1‐134	134	FM	TS432_2	34	This refinement target corresponds to domain 1 (residues 1‐134) of T0989. Remember that the original target is a homotrimer. There is a lot of room for improvement, especially in the N‐terminus
R0992	4‐110	107	TBM/FM	TS368_1	65	Residues 1–3, 111‐126 are absent from the target and deleted from the starting model
R0993s2	12‐109	98	TBM‐easy	TS246_1	51	This refinement target corresponds to second subunit of H0993 complex. The His‐tag was not observed in density, so that the chain should start with residue 12
R0996‐D4	351‐483	133	TBM‐easy	TS324_1	53	This refinement target corresponds to domain 4 (residues 351–483) of T0996
R0996‐D5	484‐604	121	TBM‐easy	TS324_1	56	This refinement target corresponds to domain 5 (residues 484–604) of T0996
R0996‐D7	709‐848	140	TBM‐easy	TS324_1	55	This refinement target corresponds to domain 7 (residues 709–848) of T0996
R0997	44‐228	185	TBM/FM	TS324_1	42	Residues 1‐43 are deleted from the starting model
R0999‐D3	866‐1045	180	TBM‐easy	TS324_1	54	This refinement target corresponds to domain 3 (residues 866–1045) of T0999. The original target is a homodimer
R1001	2‐140	139	FM	TS368_1	53	Residue 1 is absent from the target and deleted from the starting model
R1002‐D2	60‐118	59	TBM‐easy	TS023_1	66	This refinement target corresponds to domain 2 (residues 60–118) of T1002
R1004‐D2	152‐228	77	TBM‐easy	TS324_1	60	This refinement target corresponds to domain 2 (residues 152–228) of T1004
R1016	1‐203	203	TBM‐easy	TS368_1	63	This refinement target corresponds to T1016

Abbreviation: Nres, number of residues.

**Figure 1:**
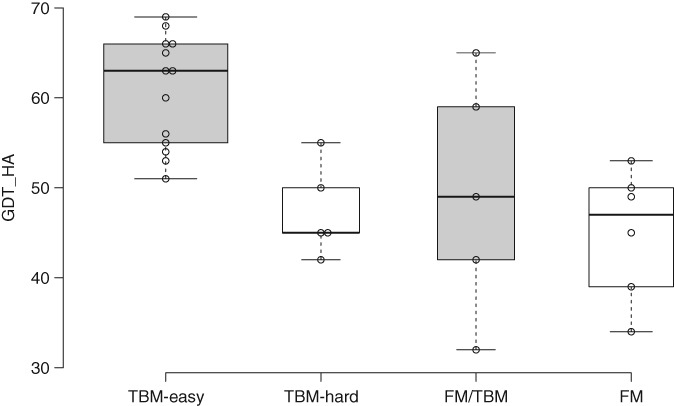
Box plot, prepared with BoxPlotR,^8^ showing the distribution of GDT_HA values seen in starting models for refinement derived from different initial modeling categories

Continuing a trend first seen in CASP12,^5^ a substantial number of refinement targets came from modeling targets initially categorized as TBM/FM (5 targets) or even FM (6 targets), with 13 from TBM‐easy and 5 from TBM‐hard (Table 1). Figure 1 shows that there is a correlation between original target category and the quality of the starting model judged by GDT_HA, but with substantial overlap between categories. In particular, the best TBM/FM starting model has a higher GDT_HA than the average TBM‐easy starting model. Although there was an attempt to choose starting models from a variety of servers to avoid bias in the initial structure prediction methods, ultimately more than half of starting models derived from just two labs. Seven starting models each were derived from models submitted by groups 324 (RaptorX‐DeepModeller) and 368 (Baker‐RosettaServer), while two more came from other Xu lab groups: one each from groups 221 (RaptorX‐TBM) and 498 (RaptorX‐Contact) (Table 1).

For each starting model, predictors were given the GDT_HA score as an indication of difficulty. They were also given some information about which residues were not visible in the experimental structure and occasionally other hints listed in Table 1, such as the presence of a Cu metal ion in R0949, which portion of the model deviates most from the experimental structure (R0981‐D5 and R0989‐D1) and what was the oligomeric state of the experimental structure (R0977‐D2, R0979, R0981 domains 3‐5, R0989‐D1, and R0999‐D3). In a first, target R0979 was an oligomeric refinement target, in which an initial trimer model was provided.

The 29 starting models covered a significant range of difficulty, as measured for instance by the GDT_HA score. This ranged from 32 to 69 (mean: 53.2, median: 53, SD: 10.2). By comparison, the 42 starting models for the refinement assessment of CASP12 had a somewhat wider range of difficulty, with GDT_HA values ranging from 24 to 78 (mean: 49.7, median: 48.7, SD: 15.0).

### 2.2 Evaluation measures

Many of the evaluation measures, particularly the utility of models for use in molecular replacement (MR) calculations, are discussed in another contribution on the topic of template‐based modeling (Croll et al., this volume). For consistency with the previous round, our primary ranking score was taken from the CASP12 refinement assessment, where relative weights of several metrics were determined by a machine‐learning algorithm trained to reproduce manual rankings.^5^ We also checked whether the ranking would have been affected by choosing the TBM ranking score used in CASP12^9^ and in CASP13 (Croll et al., this volume). Both ranking scores can readily be computed with results and tools on the Prediction Center website (http://predictioncenter.org).^10^

The refinement ranking score from CASP12 is given by the following:

$$S_{\text{CASP}12}=0.46\;z_{\mathrm{RMS}\_\mathrm{CA}}+0.17\;z_{\mathrm{GDT}\_\mathrm{HA}}+0.2\;z_{\mathrm{SG}}+0.15\;z_{\mathrm{QCS}}+0.02\;Z_{\mathrm{MP}},$$

where the *z*‐scores (SD above the mean from all predictions) for each model are computed according to the usual CASP conventions, as described in more detail in the TBM assessment (Croll et al., this volume). RMS_CA is the sequence‐dependent Cα root‐mean‐square deviation between the superposed model and target computed with local‐global alignment (LGA),^7^ GDT_HA is the high‐accuracy version of the GDT score,^7^ SG is the SphereGrinder score that measures conservation of local environment,^11^ quality control score (QCS) combines measures of the relative length, position, and orientations of secondary structure elements with Cα‐Cα distances,^12^ and MP is the MolProbity score reflecting the stereochemical quality of the model.^6^

The TBM ranking score from CASP12 is the following:

$$S_{\mathrm{TBM}}=\frac{1}{3}z_{\mathrm{GDT}\_\mathrm{HA}}+\frac{1}{9}\left(z_{\text{lDDT}}+z_{\text{CADaa}}+z_{\mathrm{SG}}\right)+\frac{1}{3}z_{\mathrm{ASE}},$$

where lDDT is the local distance difference test,^13^ a measure based on comparing all‐atom distance maps, and contact area difference, all atoms (CADaa) is a measure comparing residue contact surface areas.^14^ The accuracy self‐assessment (ASE) measure differs qualitatively in measuring not the accuracy of the model but rather the accuracy of the modelers' *estimates* of local coordinate error.^10^ Presumably because the accuracy of error estimates has not been evaluated for refinement models in previous rounds of CASP some predictors did not provide them, even though they are defined as parameters that should be included in any submitted model. To assess the impact of the ASE measure within the TBM ranking score, we also ranked models by a modified score that did not include it.

$$S_{\mathrm{TBM}\prime }=\frac{1}{2}z_{\mathrm{GDT}\_\mathrm{HA}}+\frac{1}{6}\left(z_{\text{lDDT}}+z_{\text{CADaa}}+z_{\mathrm{SG}}\right).$$

In assessing the high‐accuracy TBM category in CASP7, we introduced a MR score^15^ measuring the utility of models for solving X‐ray crystal structures by MR^16^ using our program *Phaser*,^17^ which uses likelihood‐based methods to determine the rotation and translation that places a model in the correct position in the unit cell to provide an initial atomic model for an unknown‐related molecule. The score produced by comparing the model to the experimental diffraction data, referred to as the log‐likelihood gain (LLG), can be used to assess the quality of different possible alternative models. For TBM evaluation, we computed a *z*‐score based on the LLG values found for each target for which there were experimental diffraction data. Utility for MR was tested in the evaluation of the refinement category in CASP8 through CASP10,^1^, ^2^, ^3^ though it has not been used subsequently. In CASP8 and CASP9, the translation function *z*‐score was used instead of the LLG; this is the *z*‐score (number of SD above the mean) measuring the strength of the biggest peak in a translation search with an oriented model. In CASP10, as well as in this work, a script developed by Gábor Bunkóczi was used to carry out rigid‐body refinement of a model superimposed on the experimental structure, in order to yield an LLG score without carrying out the full six‐dimensional MR search for each of the models. We are happy to provide this script, and guidance on complications that can arise in running it, on request. Further details are given in connection with the TBM assessment (Croll et al., this volume), where we also discuss plans to replace the LLG calculations with an approach that will evaluate the same model features without requiring experimental diffraction data, making it more robust and easier to use.

## 3 RESULTS

### 3.1 Group rankings

**Figure 2:**
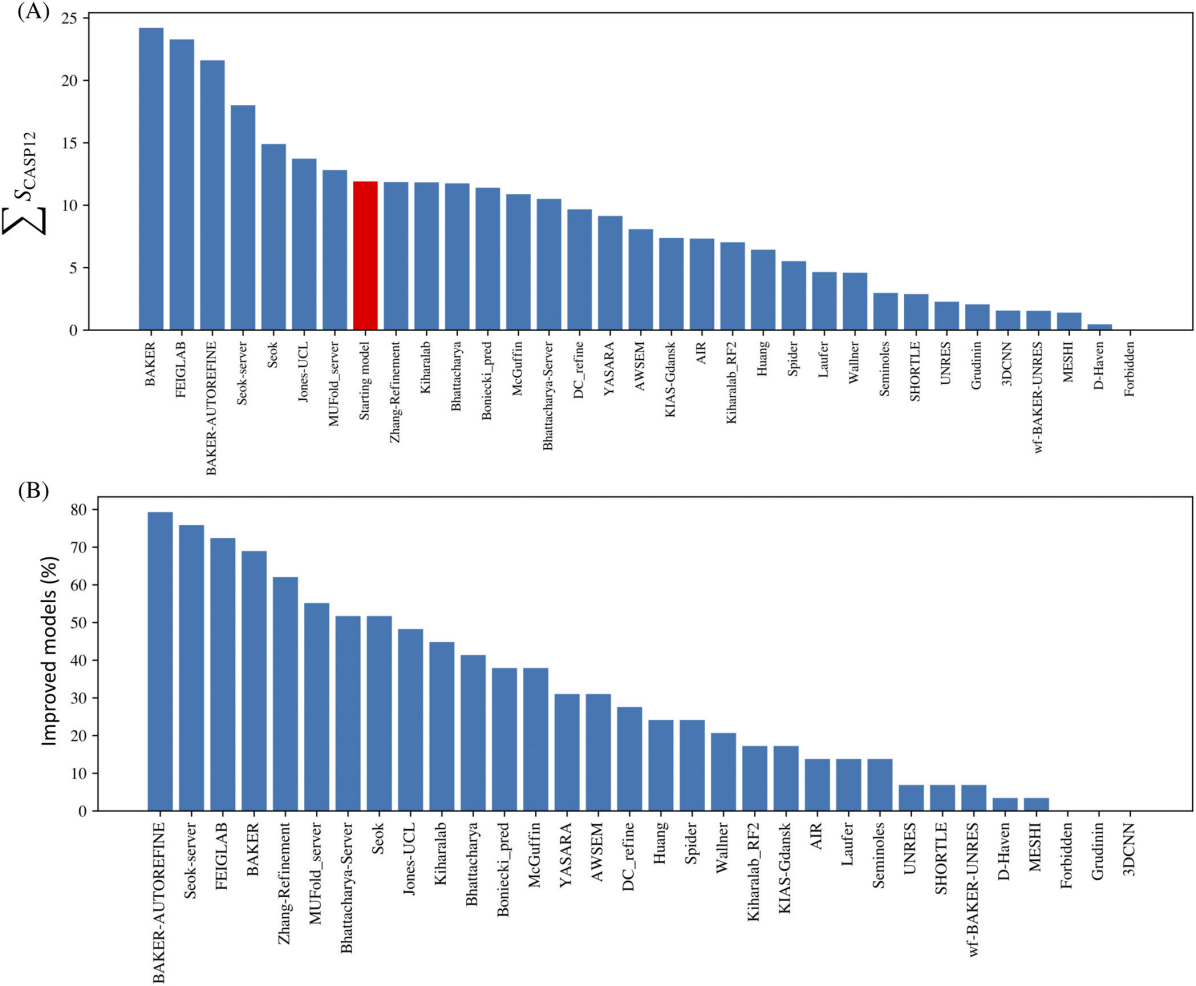
Performance of refinement groups according to default ranking score, *S*_CASP12_. A. Sum of positive *z*‐scores for all “model 1” submissions. The red bar indicates the score that would be achieved by a “naïve predictor” resubmitting each starting model. B. Fraction of times the submitted model 1 was better than the starting model for each group. It is notable that the two leading groups by this metric are automated servers

A total of 32 groups participated in the refinement category. In the group rankings, we compared their results with those that would have been achieved by a “naïve predictor,” defined as a group that simply resubmits the starting model. Figure 2 shows that, on average, predictors are still degrading the quality of the starting model by the ranking score, as the majority of groups (25 of 32) score below the naïve predictor overall while 24 of 32 degrade more models than they improve.

A number of the seven groups that ranked above the naïve predictor also did well in CASP12. The Baker group (as Baker and also as Baker‐Autorefine) were in first and third positions, having been in third position for CASP12. Feiglab, ranked second, was ranked sixth in CASP12. The Seok group (as Seok‐server and also as Seok) were ranked fourth and fifth, having ranked second (Seok) and fourth (Seok‐server) in CASP12. Jones‐UCL and MUFold_server, groups that did not appear in the top 10 ranking from CASP12, were in positions 6 and 7, respectively. Notably, two server groups were among the top seven: Seok‐server at position 4 and MUFold_server at position 6.

**Figure 3:**
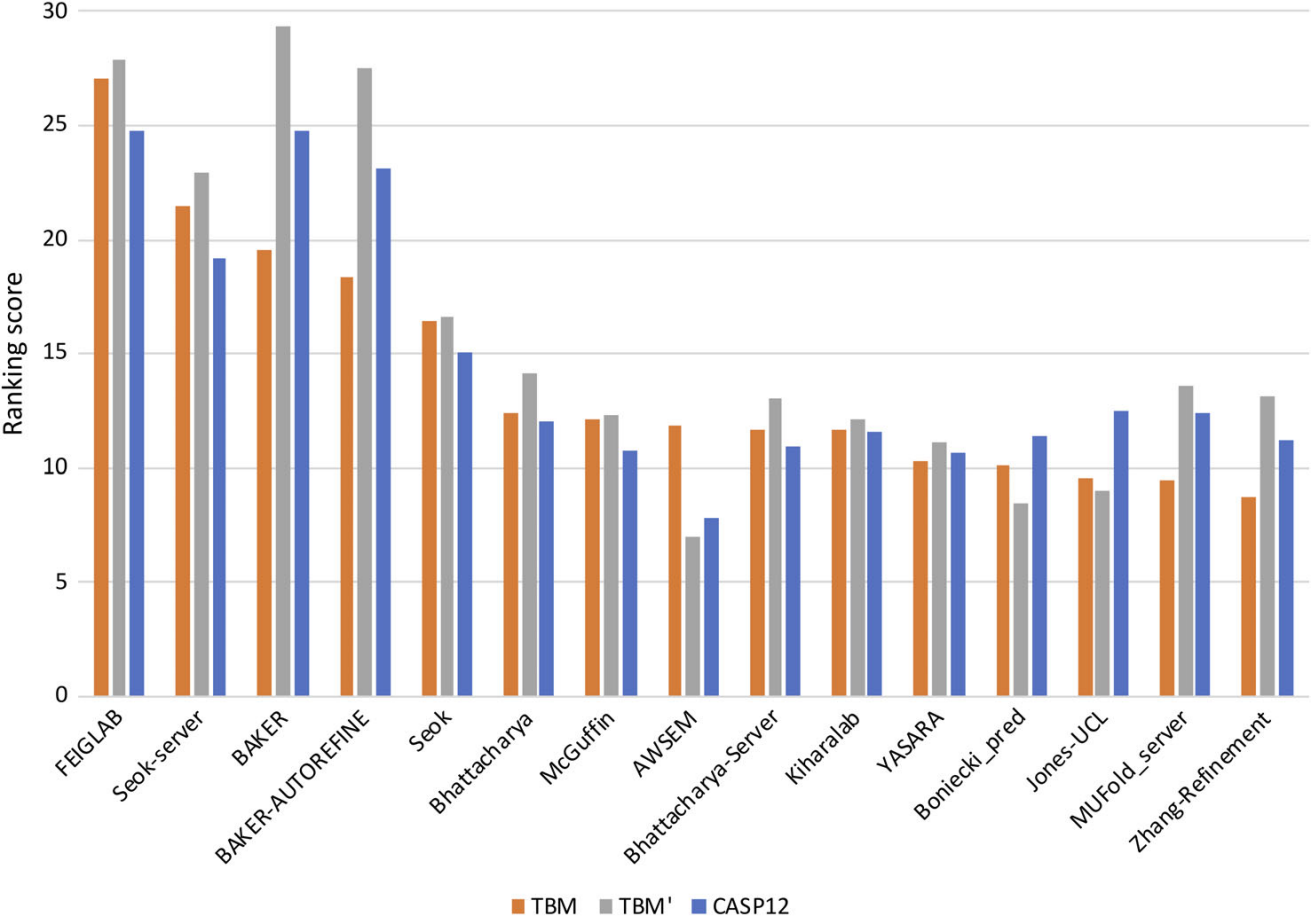
Alternative ranking ordered by the sum of *S*_TBM_, showing also the scores for *S*_TBM’_ (omitting the ASE contribution to the TBM ranking score) and *S*_CASP12_

For a more direct comparison with the TBM assessment, it is useful to see how the refinement groups would fare when judged by the *S*_TBM_ score as well. Figure 3 presents the groups in this order, showing in addition the *S*_TBM′_ score (from which the ASE metric is omitted) and the *S*_CASP12_ score. The ordering is changed significantly, although the same groups occupy the top five places (with Feiglab moving up to first place and the Baker groups down the ranking). A comparison with the *S*_TBM′_ scores shows that this difference in ranking arises primarily from the inclusion of the ASE metric, with the top five groups appearing in the same order as for *S*_CASP12_. The overall correlation between *S*_CASP12_ and *S*_TBM′_ is very high (.974), whereas the correlation between *S*_CASP12_ and *S*_TBM_ is somewhat lower (.944). However, it must be noted that this difference arises primarily because some groups did not actually provide coordinate error estimates in this category and therefore score below average for the ASE component of *S*_TBM_. Inspection of submitted coordinates shows that Baker, Baker‐Autorefine, and Zhang‐Refinement provided constant error estimates of zero or one. MUFold_server, on the other hand, provided numbers on a scale of tens to hundreds; these numbers were carried over from a step in the pipeline that used MODELLER^18^ (Junlin Wang, personal communication), which uses the B‐factor column to store violations of the target function (https://salilab.org/modeller/9.21/manual/node256.html). It seems reasonable to believe that some groups did not provide error estimates because they have not been used traditionally to assess the refinement category.

The dependence of the detailed ranking order on the choice of ranking target suggests that there is little to separate the performance of the top few groups by these criteria.

### 3.2 Assessment of progress

The assessment of progress in the refinement category is particularly difficult because refinement is a rapidly moving target. The servers generating the starting models themselves are continually improving their methods, effectively leaving refinement with fewer ways to improve a given model and just as many ways to degrade it. The improvement in server prediction methods can come, at least in principle, from lessons learned in earlier rounds of the refinement category. Furthermore, each CASP round attracts a different cohort of new groups and novel methods, not all of which will be successful. Finally, with each round the set of targets is of course completely different, inevitably introducing a large amount of noise in this measure.

**Figure 4:**
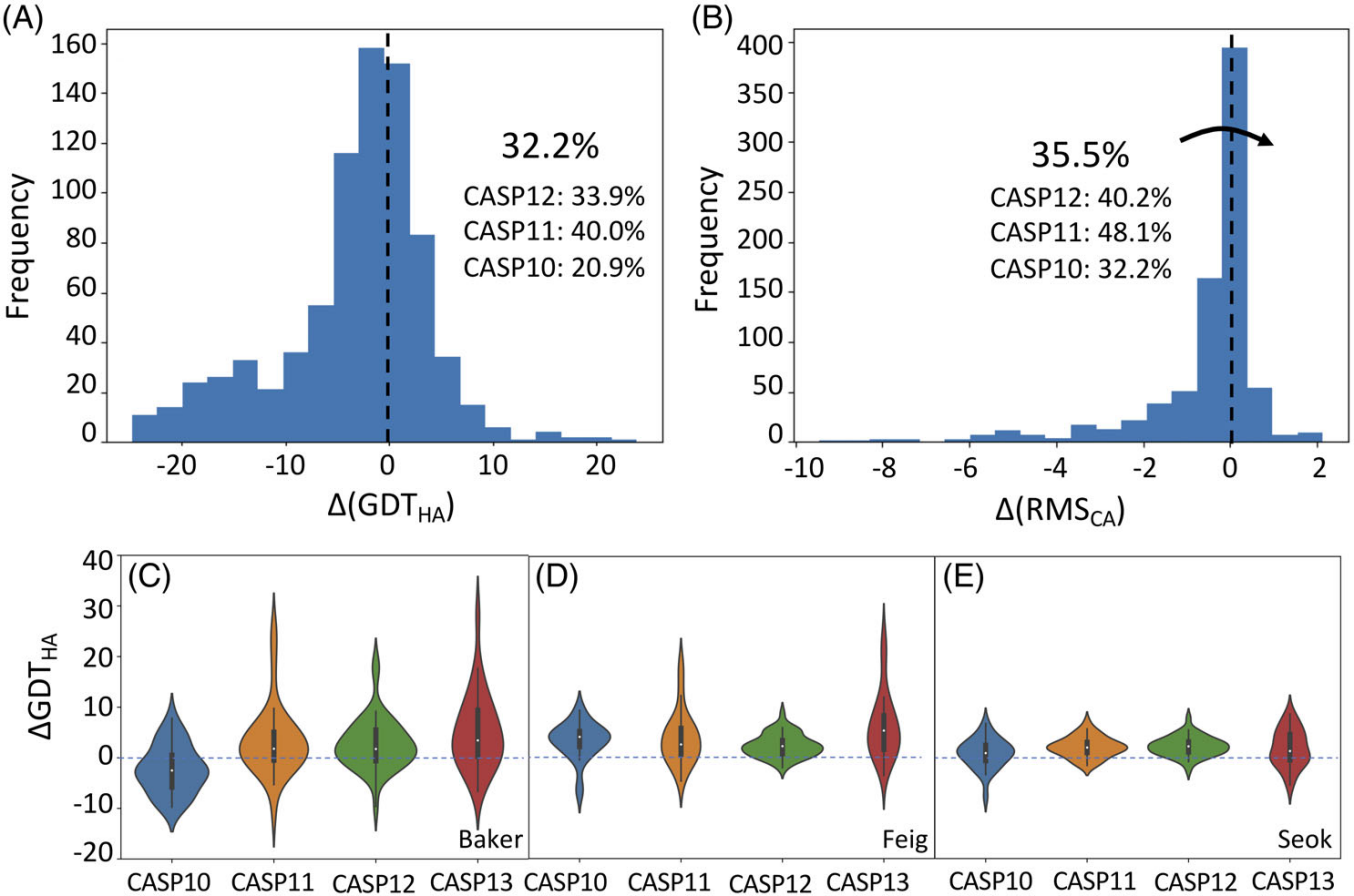
A,B. The fraction of models improved by refinement according to (A) GDT_HA or (B) RMS_CA is comparable to results in the last two CASP rounds. Each panel shows the histogram of differences from the starting model for all submitted refinement models. C‐E. Violin plots indicate that the top three human groups in CASP13 (Baker, Feig, and Seok) have achieved quite consistent improvements in GDT_HA over the last four CASP rounds, with the exception of Baker in CASP10. For the Baker and Feig groups the median improvement was higher in CASP13 than in the previous three rounds

One class of measure typically used to assess progress is the fraction of all submitted refinement models that improve on the GDT_HA and Cα RMSD metrics.^1^, ^2^, ^3^, ^5^ Histograms of the overall change in these metrics are shown in Figure 4A,B, suggesting that the progress has stalled or even reversed. However, any measure that looks at *all* submitted models will be particularly sensitive to which new groups choose to participate. In addition, it seems reasonable to consider that improved initial modeling algorithms will leave more subtle errors in the starting models, so that just continuing to improve them could be viewed as progress in itself. Looking at the top three human groups (Figure 4C‐E), we indeed see that performance according to GDT_HA has held steady or slightly increased over the last few CASP rounds.

Progress can also be assessed by looking at the performance of the top‐ranked groups as judged by the *S*_CASP12_ score. From Figure 2B, we see that 8 of 32 groups have succeeded in improving the majority of starting models. Three groups (Baker‐Autorefine, Seok‐server, and Feiglab) are able to yield better models for more than 70% of refinement targets.

### 3.3 Improvement over starting and TBM models

**Figure 5:**
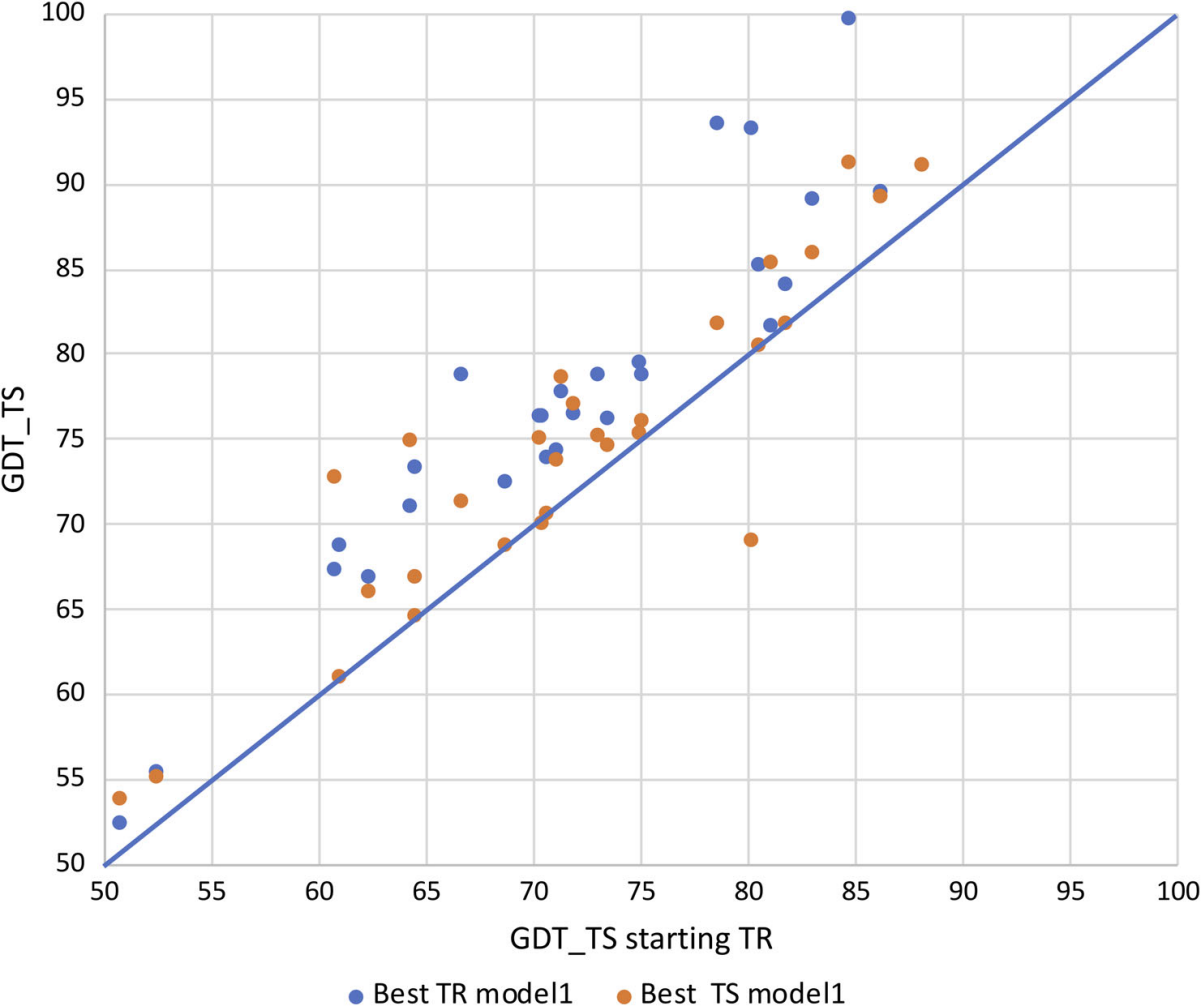
Scatter plot comparing GDT_TS scores for the best models submitted in the refinement category (TR) and in the initial models (TS) with the starting models for refinement. Orange points on the diagonal line represent cases where the starting model for refinement was the best initial model overall for that target. The single orange point below the diagonal line arises from refinement target R0986s1, where the starting model was model 4 from group A7D

The improvement that can be achieved through refinement can also be evaluated by comparing scores for the best model 1 submission with those from the starting model. This is illustrated for the GDT_TS score in Figure 5, which shows that every starting model has been improved. This improvement in scores from the starting model could be taken as an indication that the more computer‐intensive algorithms used in the refinement category truly yield better models than the algorithms used in the TBM and FM categories. Given that almost all starting models have been produced by servers, it is also possible that the involvement of human predictors is the key factor in improvement. This can be assessed by comparing the best initial model 1 from any predictor with the starting and best refined models, also shown in Figure 5. For most cases, the best refined model is better than the best initial TBM or FM model, indicating that the refinement algorithms are indeed capable of going further. However, there are a few exceptions, where a TBM or FM group could have performed best simply by submitting their model unchanged into the refinement category.

### 3.4 Geometric model quality

**Figure 6:**
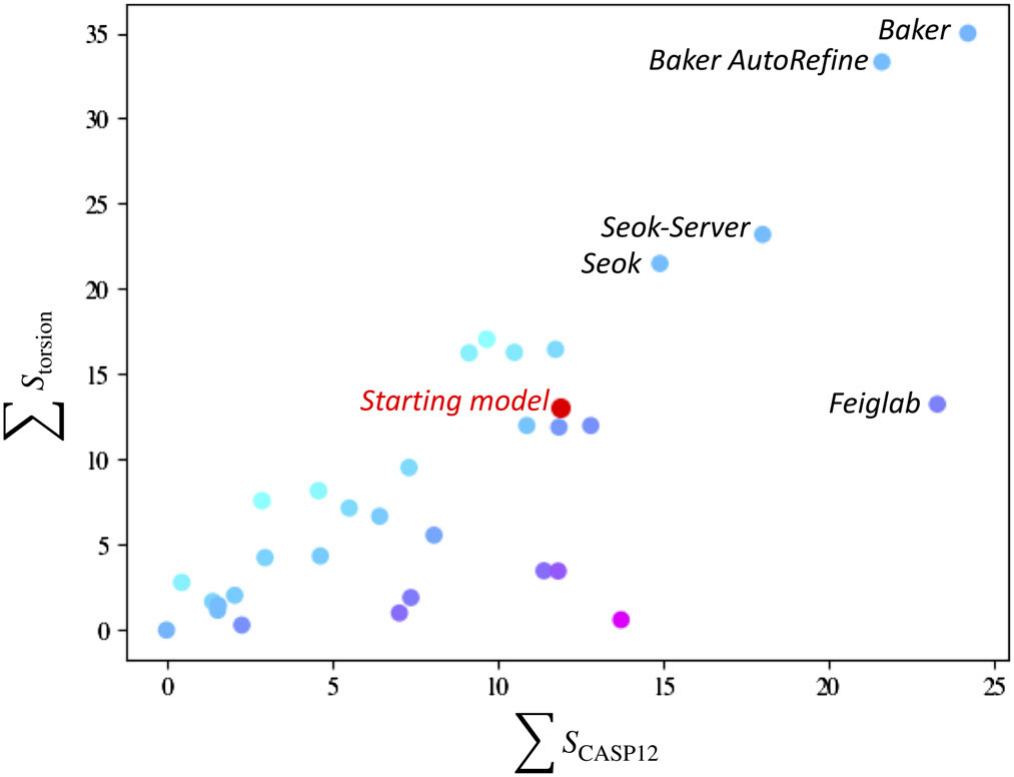
Improvements to distance‐based metrics have surprisingly low correlation with improvements to local conformation. The best results (significantly improving most models in both Cartesian and torsion space) came from the conformational search‐based methods of the Baker lab. Interestingly, the molecular dynamics‐based methods of the Feig lab led to improvements in the CASP12 rankings comparable to those of the Baker lab, while making no appreciable improvement in torsion space. The methods of the Seok lab combining knowledge‐based rebuilding with restrained molecular dynamics led to intermediate improvements by both metrics

**Figure 7:**
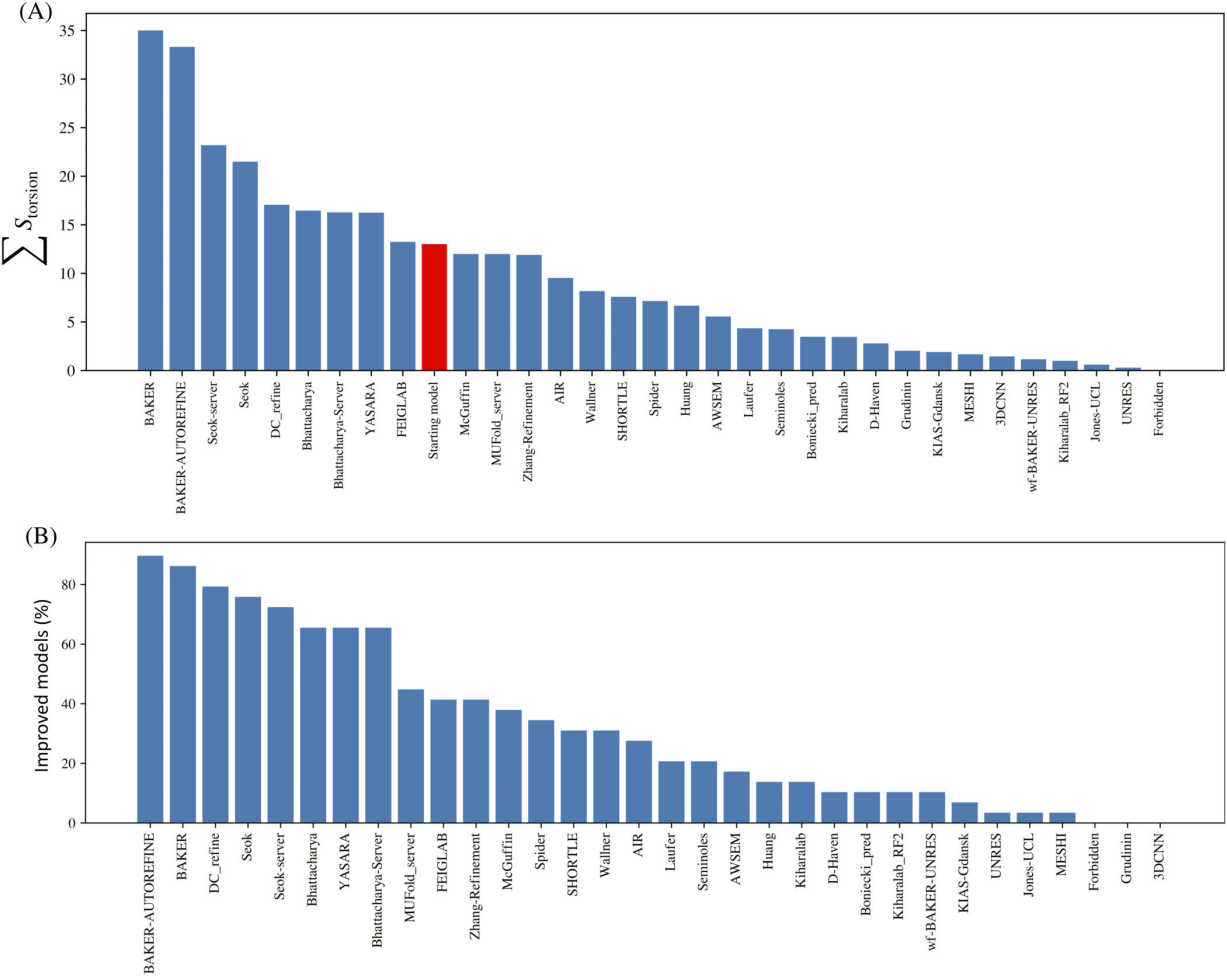
Performance of refinement groups by torsion‐based metrics. While the DC_refine, Bhattacharya, YASARA, and Bhattacharya‐Server groups do not improve the model correlation to the target in Cartesian space, each shows clear evidence of improvement in local torsional geometry

In this CASP round, alongside the standard distance‐based metrics, we explored the use of scoring in torsion space, that is, how well the local *conformation* of each model matches its equivalent in the target. In keeping with our observations of the TBM results, plotting *S*_CASP12_ against *S*_torsion_ (a weighted combination of backbone and sidechain torsion deviations as described in Croll et al., in this volume) revealed that the two measures are only poorly correlated (Figure 6). This is not surprising for models that reproduce the fold poorly, in which case the structural context required to choose the correct conformer is lacking. It was more surprising to see that the restrained molecular dynamics methods of the Feig lab led to substantial improvements in *S*_CASP12_ (enough to place them second overall by this measure)—yet, their aggregate score according to *S*_torsion_ was essentially identical to that of the naïve predictor (possible explanations for this observation are discussed below). On the other hand, the Baker‐Autorefine method that includes more aggressive conformational searching led to substantial improvements in both metrics.^23^ The Seok and Seok‐server groups (which combined molecular dynamics approaches similar to Feig with local rebuilding) yielded a somewhat more modest improvement. Overall rankings according to this metric are shown in Figure 7.

**Figure 8:**
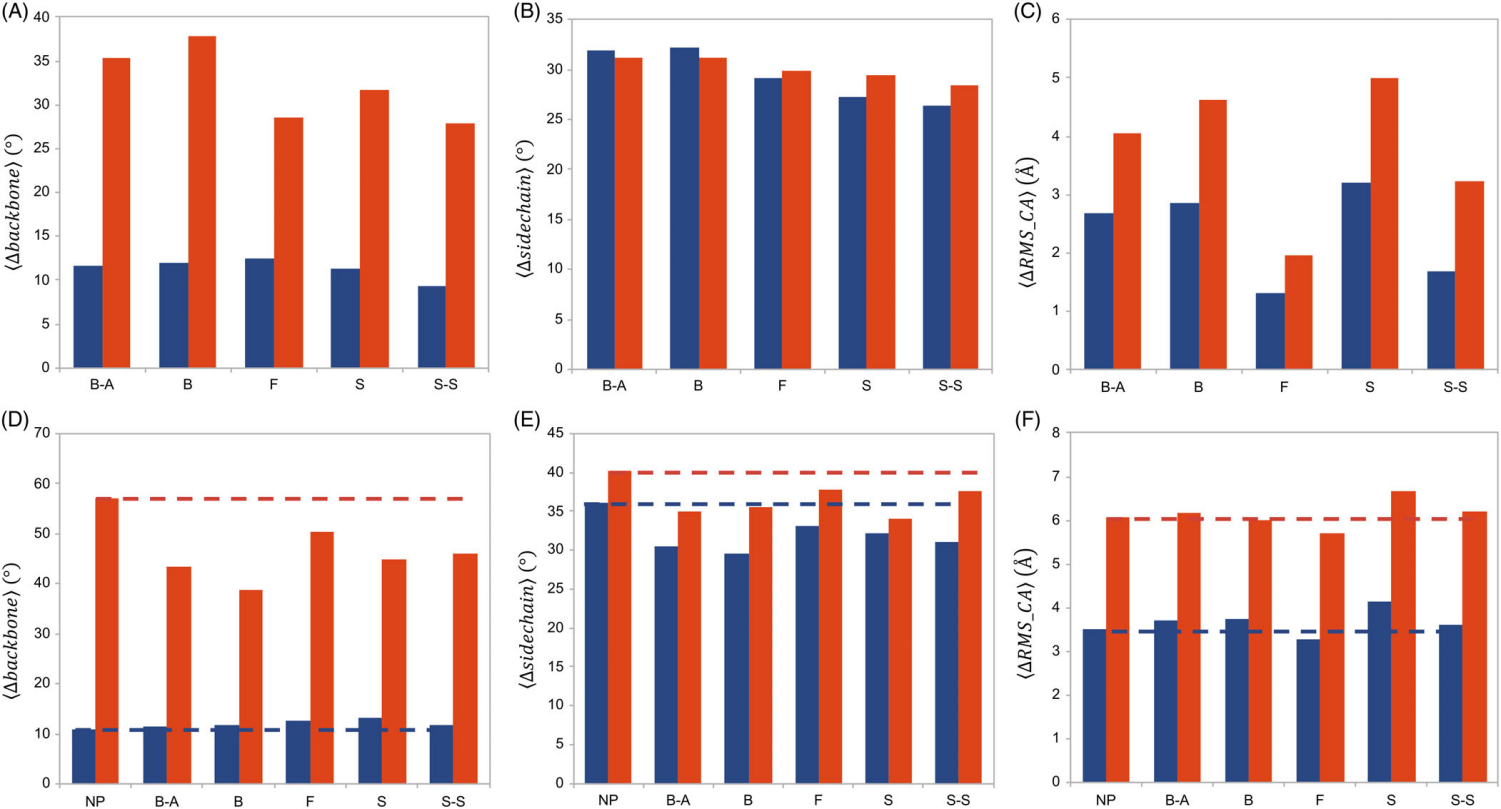
Detailed comparison of the top five groups by torsion and Cartesian metrics. Group names are abbreviated as follows: B‐A, Baker‐Autorefine; B, Baker; F, Feiglab; S, Seok; S‐S, Seok‐Server; NP, Naïve predictor. For each plot, dark blue bars are for residues where the mean backbone torsion angle error in the starting model was less than 30°, while red bars are for the remaining residues. A‐C. Average change from the starting model for (A) backbone torsions, (B) sidechain torsions, or (C) Cα positions. D‐F. Average residual error compared to the target for (D) backbone torsions, (E) sidechain torsions, or (F) Cα positions. Dashed lines indicate the thresholds for improvement over the naïve predictor. RMS_CA values were calculated after alignment of all Cα atoms in the models

Changes made to the starting models and their differences from the targets for the top five groups (by *S*_CASP12_) are explored in more detail in Figure 8. We performed separate analyses for “good” regions where the starting model essentially agreed with the target (defined as residues with average backbone torsion angle differences <30°) and the remainder where conformation differed substantially. Importantly, all five groups made only small changes to the backbone conformation in the “good” regions, suggesting that recognition and preservation of correct folds are quite robust. Changes to backbone conformation in the remaining residues were much higher, and all five groups did in fact improve overall agreement with the target by this metric. All five groups made significant changes (and improvements) to sidechain conformations. The Feig group was much more conservative in restraining Cα positions compared to the other groups. This was the only group that consistently improved the RMS_CA compared to the naïve predictor but, in agreement with the results from the *S*_torsion_ ranking, there was less improvement overall in side‐chain torsions than from the other top five groups.

### 3.5 Notable successes

**Figure 9:**
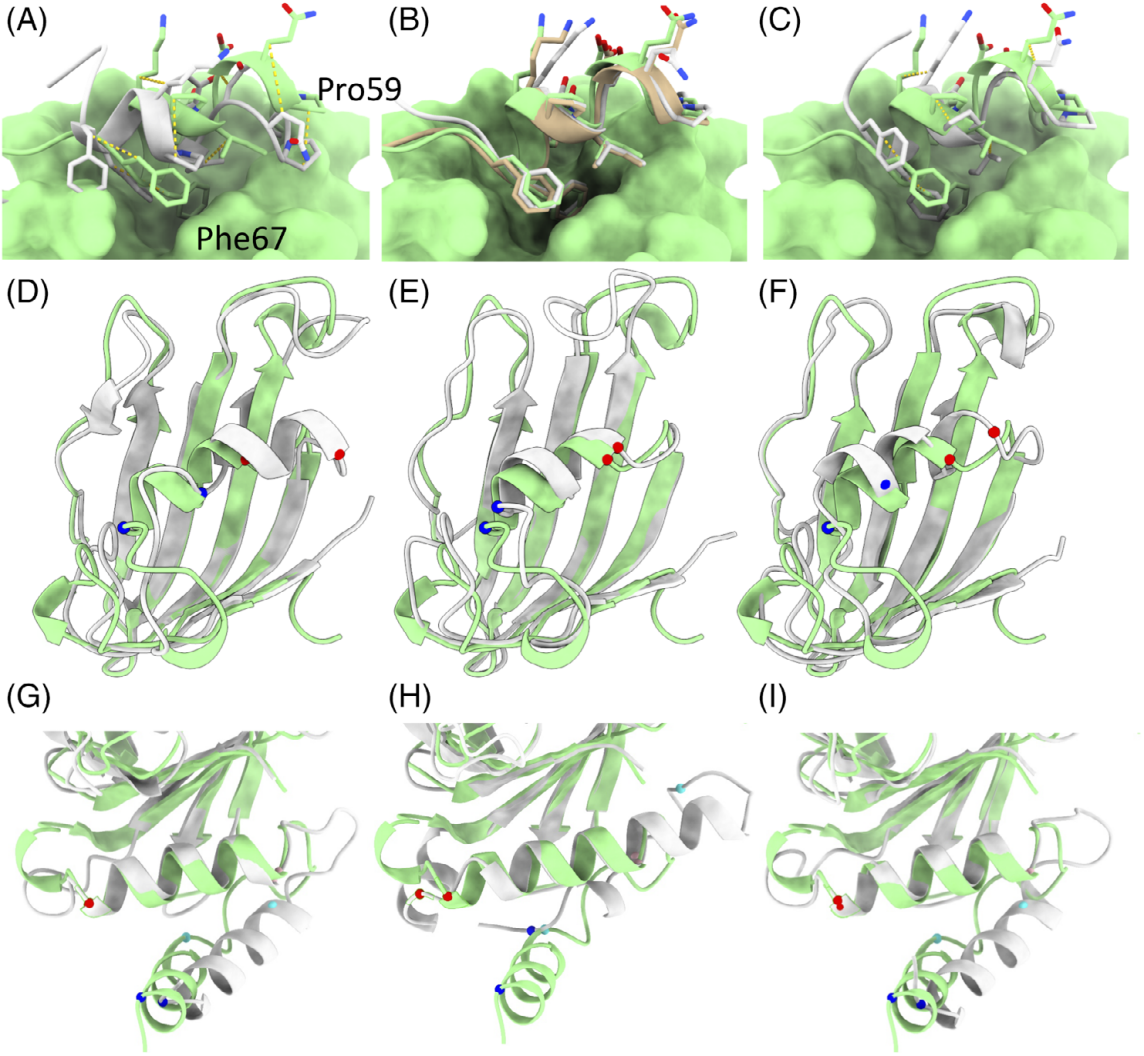
Comparison of Baker and Feig group results for three interesting cases. A‐C. Refinement target R0974s1 was a globular domain of five α‐helices, the first four of which were correct in the starting model. A. Starting model (gray) compared to target. The C‐terminal helix is tilted and shifted from its true position, with Ile62 packed into the core in place of Phe66. Equivalent C_β_ atoms are connected by dashed yellow lines. The remainder of the target is shown in surface representation. B. The Baker (tan) and Feiglab (white) models matched the target essentially perfectly. C. The Baker‐Autorefine result improved upon the starting model, but did not quite reach the target conformation. D‐F. Refinement target R0981‐D4 was a particularly notable success for the Baker group. D. While the starting model (white) closely matched the main β‐sheet in the target, the helix spanning residues 434 (Cα shown in blue) to 441 (Cα in red) was shifted about 7.5 å from its true position. E. The Baker method shifted this helix to within 2 å of its true position, and correctly predicted the conformations of the entering and exiting turns. F. The next best result (from the Feig group) brought the helix to within 5 å of the target, but added a spurious extra turn to the N‐terminus. The first 17 residues of this domain were not correctly predicted by any group, and are not shown. G‐I. The N‐terminus of R0997, in contrast, highlights a potential pitfall of the use of fragment‐based sampling methods in refinement. G. In the starting model the first helix was essentially correctly folded, but turned almost 45° from its true configuration. Additionally, the somewhat large loops flanking the second helix were poorly modeled. H. the Baker group unfolded the N‐terminal helix, added two spurious extra turns to the N‐terminus of the second helix, and partially unwrapped the C‐terminal turn of the second helix in order to fold the following loop into a helix—a significant degradation of the model quality. On the other hand, the more conservative Feig method kept the secondary structure elements correctly folded and slightly improved the disposition of the N‐terminal helix and flanking loop geometry. Cα atoms equivalent to those constituting the N‐terminus and C‐terminus of the first two helices in the target are shown, colored in blue, cyan, pink, and red in order of residue number

Two of the more impressive successes (and one less successful case study) we saw in this round are pictured in Figure 9. In each of these cases, the majority of the domain was correctly folded in the starting model (allowing for some flexibility in loops and tail regions), with a single helix shifted 5‐10 å from its true position and partially unfolded. In the case of R0974s1, the final models from Baker and Feiglab were essentially correct in core structure (including correctly modeled rotamers), differing only from the target model in the disposition of loops. On the other hand, Baker‐Autorefine made some improvement over the starting model but did not quite reach the target conformation. For R0981‐D4 the Baker and Baker‐Autorefine results were essentially indistinguishable from each other and very close to the target conformation, whereas the Feiglab result fell short (albeit closer to the target than the remaining groups). It appears likely that in this case the scale of movement in the helix triggered the Feiglab's secondary protocol for “unstable” models, using weak harmonic restraints to bias the Cα atoms to their starting positions. Interestingly, for the seemingly quite similar case of R0997 neither group was able to substantially improve upon the starting model, and the Baker group in fact significantly degraded it by refolding the first two helices into incorrect configurations.

### 3.6 Common causes of failure

**Figure 10:**
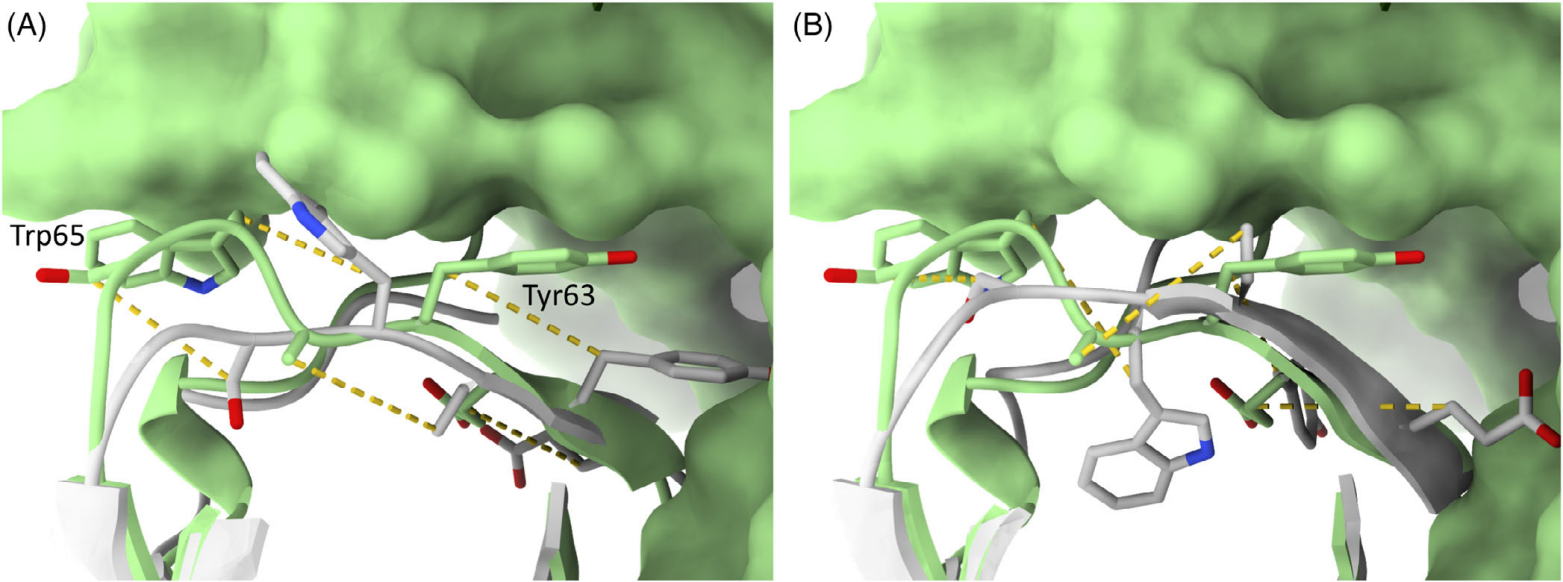
Many refinement failures arise from a lack of context. Like many targets, R1002‐D2 was a single domain excised from a larger multidomain protein. Here the target and models are shown in ribbon/stick format (with foreground loop 84‐90 hidden for clarity), while the remainder of the experimental model is shown in surface representation. A. In the experimental model (green) Trp65 and Tyr63 are buried in the interface with an adjoining domain, but shorn of this context appear to be entirely solvent‐exposed. In the starting model for refinement (white) the N‐terminal β‐strand spanning residues 59‐63 was shifted by two residues N‐terminal to its true position. B. The result from Baker‐Autorefine suggests that they correctly identified the presence of a register error here—but attempted to correct it by (sensibly, given the information available) burying these two bulky residues in the hydrophobic core, shifting the register by a single position rather than the needed two

Figure 10 is an example of perhaps the most common cause of significant failures in refinement (where all teams made the model worse): refinement targets that lack the necessary structural context. As is perhaps inevitable given the trend in experimental structural biology toward tackling larger and larger complexes, very few of the refinement targets this year exist in nature as isolated single domains. Many in fact form symmetric homomultimers; others are involved in specific protein:protein interactions; still others (such as this example) form part of a larger multidomain protein. In such cases, it is common for some portion of the target to simply not make sense in isolation: an extended strand or hairpin which is only stabilized by interactions with an adjacent domain; large, solvent‐exposed hydrophobic patches; or (as here) a tryptophan and tyrosine apparently fully solvent exposed on an unstructured loop. The challenge in interpreting this is further compounded by the fact that in the starting model the immediately preceding β‐strand is out of register by two residues (the remainder of the model is essentially correct). Baker‐Autorefine correctly identified that this strand required rebuilding, in the course of which the sequence register was changed—but in what would normally be a quite sensible move it incorrectly opted to bury the “solvent‐exposed” Tyr63 and Trp65. This led to a one‐residue shift rather than two‐residue shift in the offending β‐strand, while the introduction of two bulky sidechains into the hydrophobic core significantly disrupted the packing of the domain, resulting in an arguably worse model than those from other groups that did not change the sequence register in this region.

### 3.7 MR model quality

**Figure 11:**
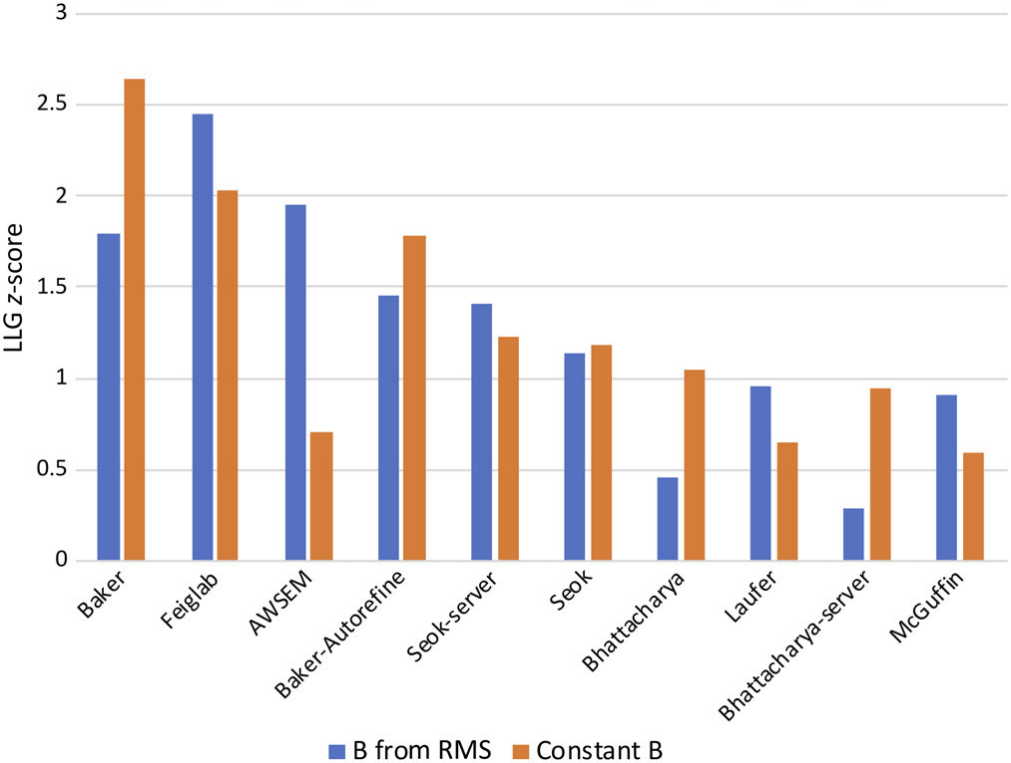
MR LLG *z*‐scores for top groups, sorted by the maximum *z*‐score obtained either with error‐weighted or unweighted models. Note that, although the LLG values will be unchanged when groups provide constant coordinate error estimates, the *z*‐scores become lower because of improved performance from other groups

Diffraction data were available for 11 of the 31 refinement targets. LLG scores were computed using *Phaser* 19 both with and without error weighting, as discussed above. To put the results from different targets on the same scale, *z*‐scores were computed, carrying out the calculation separately for LLG values obtained with and without error weighting. Computing the *z*‐scores separately for each target helps to correct for differences among the targets in quality of diffraction data, the number of copies of a target in the asymmetric unit of the crystal, and the presence of unmodeled components, as discussed in more detail in the paper on TBM assessment (Croll et al., this volume). Groups were ranked, as shown in Figure 11, by mean *z*‐score. There is considerable overlap between the top groups by this ranking and *S*_CASP12_, with Baker, Feiglab, Baker‐Autorefine, and Seok‐server all appearing in the top five of both lists. However, the group AWSEM, which is in position 17 by *S*_CASP12_, appears in third place by the MR ranking, but only when the LLG score computed by using error weighting is considered. This is a very striking example of how much value can be added to the MR calculation when good estimates of coordinate error can be provided. Feiglab moves into first place when error weighting is considered but Baker, which failed to provide error estimates, drops from first to third in the ranking.

**Figure 12:**
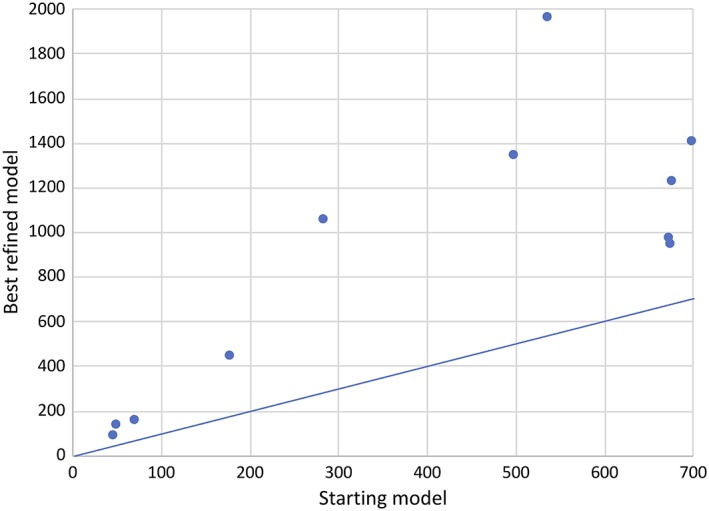
Scatter plot comparing the increase in LLG obtained by adding the starting model to a background comprising the rest of the crystal structure with that obtained using the best refined model

**Figure 13:**
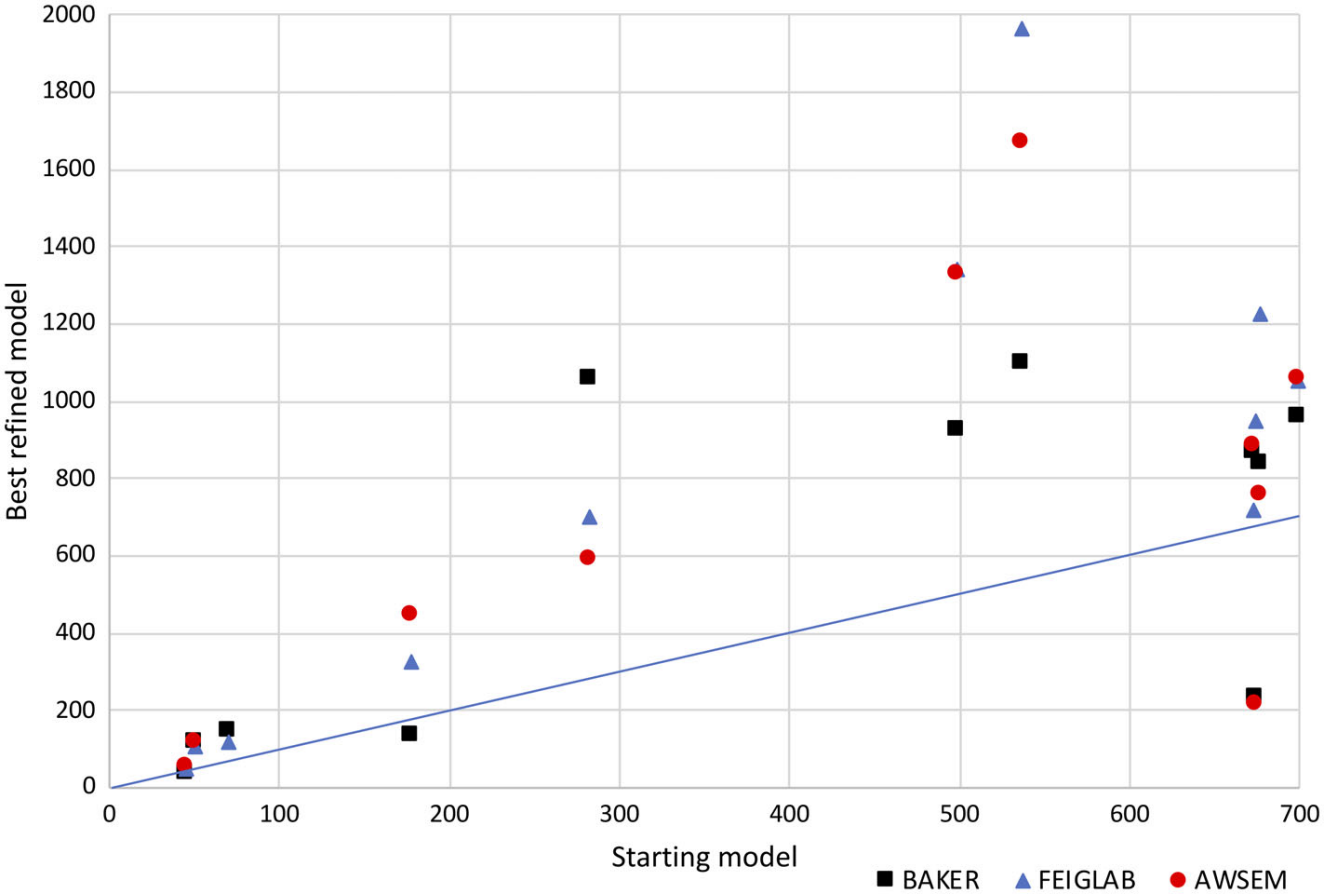
Scatter plot comparing the increase in LLG obtained by adding the starting model to a background comprising the rest of the crystal structure with that obtained using the best refined model from each of the three top‐ranked groups

In every case, at least one model gives an improved LLG score compared to the starting model (Figure 12). Figure 13 shows that the top groups improve on the starting model in most, but not all, cases.

## 4 DISCUSSION

Broadly speaking, the most successful refinement groups by both the traditional and proposed new measures (ie, the Baker and Seok groups, which achieved significant improvements in both gross and local conformational match to the target) combined physics‐based force fields with some level of directed conformational search, relying on MD primarily to “relax” selected conformations to their local minima over short timeframes. On the other hand, the more MD heavy methods of the Feiglab, while achieving significant improvements according to the primary *S*_CASP12_ target, yielded little to no improvement in local torsional geometry. At first sight, this result seems surprising, and it is worth exploring possible explanations.

The beginning and end of the Feig protocol involves the use of LocPREFMD^20^ to regularize model geometry. In brief, this involves the following key steps: (a) add missing atoms; (b) rebuild nonproline *cis* peptide bonds to *trans*; (c) rebuild badly clashing ring sidechains into nonclashing conformations; (d) minimize with gradually increasing Cα position restraints; (e) rebuild rotamer outliers; (f) equilibrate at gradually increasing temperatures; (g) minimize selected snapshots from equilibrium ensemble; and (h) choose final model based on MolProbity score and Cα RMSD to original model. While these steps (in particular, the active rebuilding of problematic sidechains and *cis* peptide bonds) in general appear sensible, a potentially serious problem arises from the use of artificially strengthened CMAP potentials in the MD force field. CMAP potentials are designed to adjust the potential energy of the peptide backbone as a function of *φ* and *ψ* to more closely recapitulate observed conformations (ie, the distribution of residues on the Ramachandran plot).^21^ Strengthening these terms has the effect of pushing residues in marginal or disallowed conformations toward the nearest “favored” region of Ramachandran space. As has been learned in the field of experimental model building, such “Ramachandran restraints” are often counterproductive.^22^ The problem in essence is that in any physically realistic force field, the *nearest* favored conformation to a stable outlier is rarely the *correct* conformation. The more common scenario is that the offending residue's backbone is sterically trapped in a conformation where one or both of its flanking peptide bonds is flipped close to 180° from its true low‐energy state. In such situations, the net effect of Ramachandran restraints is to push the conformation “uphill” into a high‐energy state which, while achieving a lower MolProbity score, is not necessarily any more correct than the original. Because of the connected nature of the polypeptide backbone, this further tends to push errors into the neighboring residues. A possibly more successful strategy might be to use the appearance of Ramachandran outliers in the same manner as rotamer outliers: as cues indicating the likely need for more aggressive local rebuilding.

The question of how to handle refinement of single domains culled from their context in larger complexes is a challenging one with no easy answers. Even if not critical to the stability of the domain's fold per se, interdomain contacts often stabilize specific conformations of otherwise‐flexible loops and/or involve bulky/hydrophobic residues (eg, Figure 10). Removing the context causes such residues to appear solvent exposed, leading to large conformational changes in MD simulations and confusing conformational search algorithms. Providing the *true* (experimental) context is not a satisfying solution—not only is this unrealistic in terms of most real‐world uses, but this would also allow most targets to yield only a single domain for refinement. One possible solution would be to provide the entire server model as starting coordinates, with instructions specifying which portion is to be focused on for refinement.

## 5 CONCLUSIONS

Progress in the model refinement task is difficult to measure: it inevitably becomes more difficult from one round of CASP to the next, as the predictors providing the starting models become increasingly sophisticated and leave only more subtle errors that are more difficult to address. By one of the measures that had shown improvements in past rounds of CASP (the fraction of all submitted models that improve on the starting model), progress in the general refinement community might appear to have stalled or even reversed. We feel that this conclusion would be too pessimistic: the fact that some of the refinement groups are still consistently able to improve on the best of the models provided in the initial predictions shows that the best refinement methods are matching the more easily measured improvements in the initial modeling methods.

For consistency, we used the score developed for CASP12 as our primary ranking score. However, we believe that in the future this should incorporate metrics that make greater demands, including agreement with main‐chain and side‐chain torsion angles. Even though the TBM and FM predictors are now largely providing coordinate error estimates, it seems that many participants in the refinement category fail to do so because this has not typically been used in assessment. Because good error estimates are, in fact, an essential part of a useful model, we find it unfortunate that they have been neglected traditionally in this category and strongly believe that they should be required here as well. It might also be interesting to evaluate, along with the refined model, some annotation of which parts of the model the predictor believes have been improved.

By convention, the starting models for refinement come with hints about the target. Some of these (such as the oligomeric state of the molecule or the presence of a ligand or bound metal ion) are facts that would frequently be known in a real‐life modeling scenario. On the other hand, one would be unlikely to know the GDT_HA score of an intermediate model, yet this can be (and is) used to decide between more and less conservative approaches. The starting model is almost always the best server model provided in the initial modeling round. In principle, the knowledge of which server models were *not* chosen could be exploited, though it is difficult to know if it is. Perhaps a more random choice from among the better server models should be used.

Finally, the nature of available targets in this round of CASP reflected the move in structural biology toward larger assemblies, assisted in part by recent dramatic improvements in the capabilities of cryo‐EM. A number of the targets were, in fact, components from very large assemblies determined by cryo‐EM. As a result, many of the evaluation units for TBM and refinement targets chosen from them are small components divorced from their structural context. In a number of cases, knowledge of the context would be essential to making an accurate prediction. Some consideration should be given to how refinement targets can be chosen and presented to provide a better indication of their context.

## CONFLICT OF INTEREST

The authors declare no potential conflict of interest.
